# Designer drugs

**DOI:** 10.1093/labmed/lmad087

**Published:** 2023-09-29

**Authors:** Amadeo J Pesce, Kevin Krock

**Affiliations:** Precision Diagnostics, San Diego, US; Precision Diagnostics, San Diego, US

Designer drugs are synthetic compounds developed to mimic the physiologic effects of other abused drugs. Many designer drugs are chemically similar to other abused drugs but are modified to avoid being classified as illegal. Moreover, they are often altered in ways that render them undetectable by conventional drug screening tests. Most designer drugs try to imitate opiates or cocaine, ecstasy, and other stimulants.

Unfortunately, it is also possible to overdose on designer drugs.^1^ The unpredictable pharmacology of these synthetic drugs puts users at risk of dangerous side effects, including overdose and death.^2^ One of the greatest challenges associated with detecting designer drugs is the fact that they are developed in secret. Therefore, the ingredients, chemical structure, and potency of the drugs are largely unknown. The US Drug Enforcement Administration recognizes 7 different types of designer drugs: cannabinoids, phenethylamines, phencyclidines (or arylcyclohexamines), tryptamines, piperazines, pipradrols, and N-ring systems. Most laboratories using liquid chromatography–tandem mass spectrometry (LC-MS/MS) for definitive drug testing are targeting well-known drugs and are not configured to detect or quantify these synthetically modified drugs.

These designer drugs can be detected in urine using newer analytical methods such as time of flight (TOF) or Orbitrap mass spectrometry. These technologies require more sophisticated equipment and are more labor intensive than definitive methods such as LC-MS/MS. Currently, the reimbursement for the Current Procedural Terminology (CPT) code applied to drug screening is only $60. This reimbursement was established for the historic immunoassay screens and the older ToxiLab thin-layer chromatography method. The newer technologies identify over 1000 possible drugs, including designer drugs, but it is a labor-intensive approach to drug testing.^3^ The current reimbursement is too low to be financially viable for a commercial or hospital laboratory seeking to use more advanced technologies to detect drugs. There is a need to create a CPT code that pertains to use of the newer technologies for toxicological analysis so laboratories that wish to include designer drugs in their drug testing panels can be reasonably reimbursed. We believe there are enough data on overdose deaths from designer drugs to warrant testing for them in specific patient populations, such as those in drug rehabilitation facilities. Reimbursement would require medical necessity, and we propose the following guidelines:

Rationale (medical necessity for new CPT code and reimbursement):

1. Patient is in a pain clinic or drug rehabilitation facility
2. Clinical signs of impairment
3. Positive immunoassay screen, but negative by the usual targeted LC-MS/MS analysis of common drugs
4. Suspicion of designer drug use
5. Physician (provider) requests further testing

As an example, there are numerous fentanyl derivatives available to patients that we cannot identify with our current targeted testing, and a substantial portion of current opioid deaths are due to fentanyl.^4^ The use of TOF-based analytical methods would enable better detection of designer drug use. Current reimbursement makes this unworkable.

## Abbreviations

LC-MS/MS: liquid chromatography–tandem mass spectrometry
TOF: time of flight

## About the Author

Amadeo J. Pesce, PhD

Amadeo J. Pesce, PhD, DABCC, is the Laboratory Director at Precision Diagnostics LLC in San Diego, CA. He has 45 years of experience in toxicology testing. He also holds the position of adjunct professor in the Department of Pathology and Laboratory Medicine at the UCSD School of Medicine. He obtained his bachelor’s degree from MIT and his doctorate from Brandeis University. He was a National Institutes of Health postdoctoral fellow at the University of Illinois (Urbana) under the direction of Dr Gregorio Weber. Dr Pesce has published or edited more than 20 books or monographs in the field of Clinical Chemistry and is the author or coauthor of more than 270 papers in the field of Clinical Chemistry and Clinical Toxicology.

Dr Pesce has been the recipient of several awards, including Established Investigator, American Heart Association New York, the Alvin Dubin Award from National Academy of Clinical Biochemistry, the Diploma of Honor from the Association of Clinical Scientists, and the Irving Sunshine Award from the International Association of Therapeutic Drug Monitoring and Clinical Toxicology.

Dr Pesce and his staff have been performing urine drug testing for many years, most recently focusing on the needs of pain patients and those undergoing addiction treatment.
